# Complete spectrum of adverse events associated with chimeric antigen receptor (CAR)-T cell therapies

**DOI:** 10.1186/s12929-023-00982-8

**Published:** 2023-10-21

**Authors:** Chieh Yang, John Nguyen, Yun Yen

**Affiliations:** 1 Department of Internal Medicine, School of Medicine, University of California Riverside, Riverside, CA USA; 2 Covina Discovery Center, Theragent Inc., Covina, CA USA; 3 College of Medical Technology, Taipei Medical University, Taipei City, Taiwan

**Keywords:** Chimeric antigen receptor (CAR)-T cell therapies, Cytokine release syndrome, Immune effector cell-associated neurotoxicity syndrome, Macrophage activation syndrome

## Abstract

Chimeric antigen receptor (CAR)-T cell therapies have been approved by FDA to treat relapsed or refractory hematological malignancies. However, the adverse effects of CAR-T cell therapies are complex and can be challenging to diagnose and treat. In this review, we summarize the major adverse events, including cytokine release syndrome (CRS), immune effector cell-associated neurotoxicity syndrome (ICANS), and CAR T-cell associated HLH (carHLH), and discuss their pathophysiology, symptoms, grading, and diagnosis systems, as well as management. In a future outlook, we also provide an overview of measures and modifications to CAR-T cells that are currently being explored to limit toxicity.

## Background

In recent years, relapsed or refractory hematological malignancies have been treated with chimeric antigen receptor (CAR-)T cells with unprecedented success. There are currently six US FDA-approved CAR-T cell therapies (Table 1) [1], of which four target CD19. Anti-CD19 CAR-T cells are used in B cell malignancies, such as relapsed or refractory follicular lymphoma, large B cell lymphoma (LBCL), mantle cell lymphoma (MCL), and precursor B cell acute lymphoblastic leukemia (ALL). The other two CAR-T therapies target B cell maturation antigen (BCMA) and are approved for the treatment of relapsed or refractory multiple myeloma.

**Table 1:** Summary of current FDA-approved CAR-T cell therapies

Proper Name	Tradename	STN#	Target antigen	Manufacturer	Indications	Co-stimulatory domain
idecabtagene vicleucel	ABECMA	125736	BCMA	Celgene Corporation, a Bristol-Myers Squibb Company	Relapsed or refractory multiple myeloma	4-1BB
lisocabtagene maraleucel	BREYANZI	125714	CD19	Juno Therapeutics, Inc., a Bristol-Myers Squibb Company	Large B-cell lymphoma (LBCL), including diffuse large B-cell lymphoma (DLBCL) not otherwise specified (including DLBCL arising from indolent lymphoma), high-grade B-cell lymphoma, primary mediastinal large B-cell lymphoma, and follicular lymphoma grade 3B	4-1BB
ciltacabtagene autoleucel	CARVYKTI	125746	BCMA	Janssen Biotech, Inc	Relapsed or refractory multiple myeloma	4-1BB
tisagenlecleucel	KYMRIAH	125646	CD19	Novartis Pharmaceuticals Corporation	Relapsed or refractory follicular lymphoma after two or more lines of therapy	4‐1BB
brexucabtagene autoleucel	TECARTUS	125703	CD19	Kite Pharma, Inc	Relapsed or refractory mantle cell lymphoma (MCL), relapsed or refractory (r/r) B-cell precursor acute lymphoblastic leukemia (ALL)	4-1BB
axicabtagene ciloleucel	YESCARTA	125643	CD19	Kite Pharma Inc	Refractory or relapsed large B-cell lymphoma	CD28

**Fig. 1:**
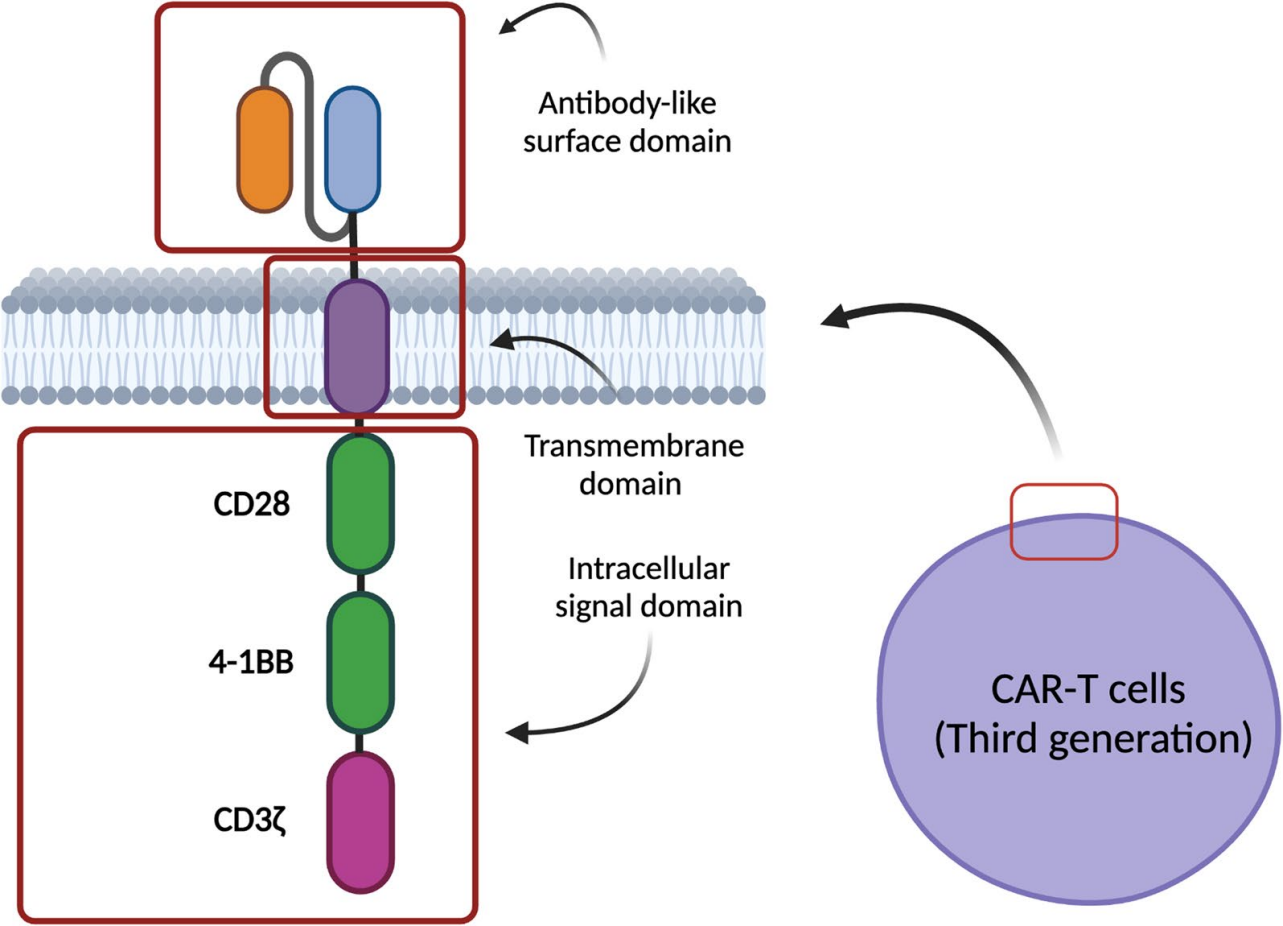
Structure of CAR-T cells. Created with BioRender.com

Chimeric antigen receptors (CARs) are synthetic immunoreceptors that combine an antibody-derived antigen-binding extracellular domain with activatory intracellular signaling domains of the CD3/T cell receptor (TCR) complex and T cell co-stimulatory receptors (Fig. 1) [2–4]. Thus, T cells can be engineered with CARs to recognize virtually any cell surface antigen. The first generation of CARs possessed the CD3ζ intracellular domain as their sole signaling domain, and this design was able to induce tumor cell killing in vitro but performed poorly in vivo. The addition of a signaling domain from a T cell co-stimulatory receptor (such as CD28 or 4-1BB) marked the second generation of CARs, providing CAR-T cells with enhanced activation, expansion, and persistence in vivo [2, 5–8]. All currently approved CAR-T cell therapies utilize second-generation CARs, and they target the antigens CD19 and BCMA due to specificity and expression restricted to the B cell lineage and plasma cells, respectively [2, 9]. Further iterations are being studied, such as third-generation CARs containing two co-stimulatory domains, e.g. a combination of CD28 and 4-1BB [6–8]. Furthermore, additional tumor-associated target antigens are being evaluated, including CD22, CD33, CD70, CD123, CD138, CD171, HER2, EGFR, B7-H3, claudin 6, gp120, GPRC5D, PSMA, and mesothelin [6, 10–14]. In attempts to reduce tumor escape through antigen loss, simultaneous targeting of multiple antigens, such as a combination of CD19 and CD22, has also been considered [15, 16].

Despite favorable clinical response rates, the manufacturing of CAR-T cells is as complex as for any other adoptive cell therapy, resulting in logistical challenges and high cost of these treatments [2]. Leukapheresis is performed on the patients to isolate autologous leukocytes, from which T cells are enriched in the manufacturing facility. In a process spanning two to four weeks, these T cells are activated, retrovirally or lentivirally transduced with the CAR gene, and expanded to yield sufficient doses of CAR-T cells to re-infuse into the patient after conditioning chemotherapy. In addition to logistics, costs and turnaround time, the multifaceted impacts of CAR-T cells on the human body have to be taken into consideration [19, 20]. In this review, we will discuss our current understanding of pathophysiology and management strategies for the major and most frequent CAR-T cell-related adverse events: Cytokine release syndrome (CRS), immune effector cell-associated neurotoxicity syndrome (ICANS), and hemophagocytic lymphohistiocytosis or macrophage activation syndrome (HLH/MAS).

### CAR-T cell-related adverse events

**Fig. 2:**
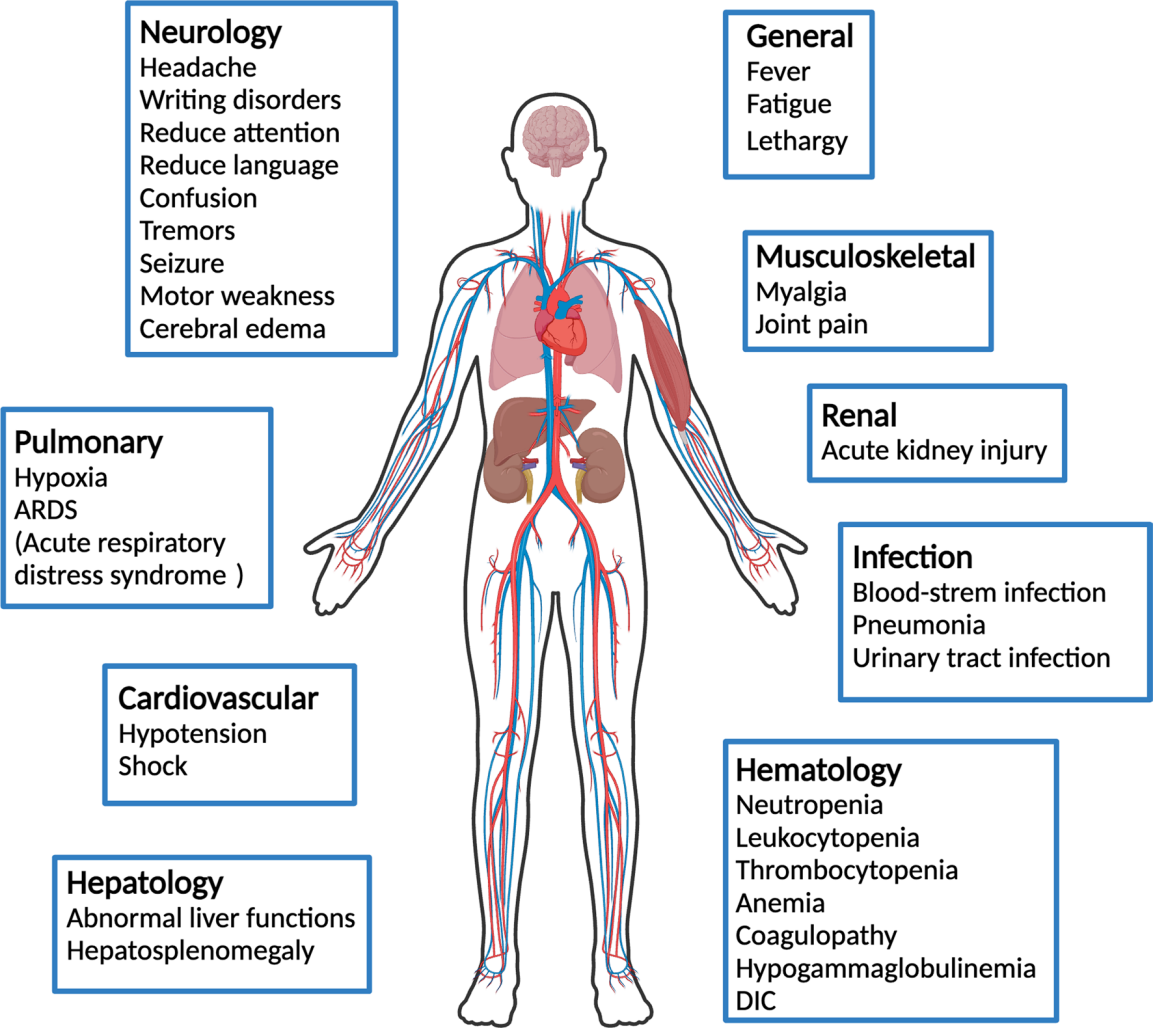
Simplified CAR-T therapy’s adverse effects. Created with BioRender.com

Since many mechanistic aspects of their pathophysiology are still poorly understood, the diagnosis and treatment of CAR-T cell-related adverse events pose unique challenges. These adverse effects span a broad range of severities and manifestations and may involve multiple organ systems (Fig. 2), similar to immune-related adverse events (irAEs) that are known to occur upon use of immune checkpoint inhibitors [21, 22]. On-target off-tumor effects, whereby healthy cells expressing the target antigens are attacked by the CAR-T cells, are common, but in the cases of therapies targeting CD19 and BCMA they are manageable and well-tolerated [20]. There have been reports of allergic reactions and metabolic abnormalities such as tumor lysis syndrome after CAR-T cell infusion [20, 23–25]. However, CRS, ICANS, and HLH/MAS are regarded as the most dominant CAR-T cell-related toxicities.

**Fig. 3:**
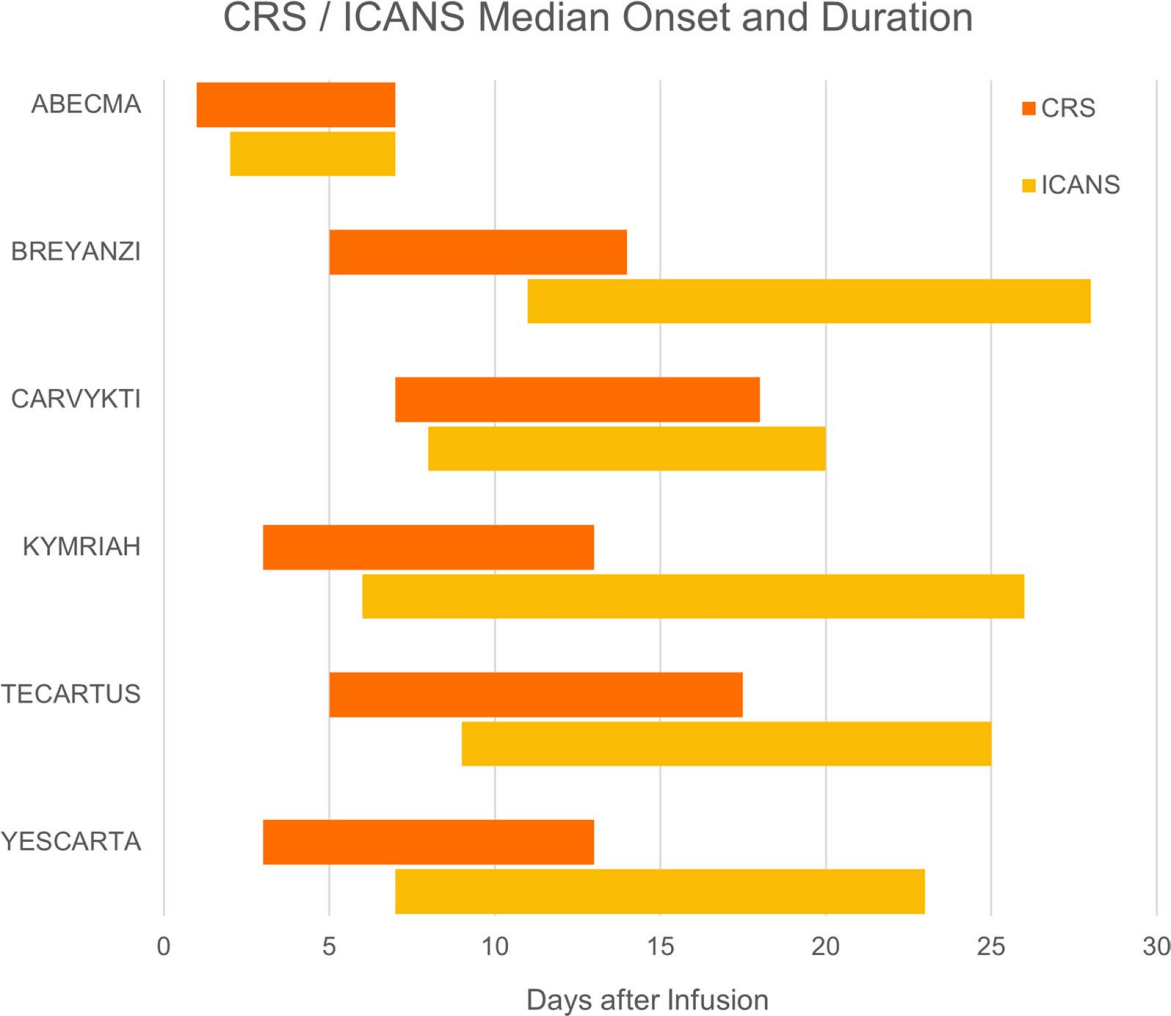
Median onset and duration of CRS and ICANS after six FDA-approved CAR-T cell therapies

CRS and ICANS are the most frequent adverse events of CAR-T cell therapies [26–31]. In clinical trials of the six currently US FDA-approved CAR-T products, CRS had an incidence of between 49 and 95%, with 1–24% for grade ≥ 3 CRS. ICANS occurred in 12–60% of the patients, with grade ≥ 3 ICANS in 3–50% (Table 2) [26–31]. Differences in grading systems likely contributed to the large variability in recorded frequencies of adverse events between clinical trials, making it difficult to draw direct comparisons. Nevertheless, a common pattern is the earlier median onset of CRS within the first week after CAR-T cell infusion, compared to ICANS which tends to also have a longer average duration (Fig. 3) [26–31]. Despite the high incidence of CAR-T cell-related adverse events, a meta-analysis estimated treatment-related death at only 1% [36].

**Table 2:** Summary of incidence of CRS and ICANS after CAR-T cell therapies

Name of CART therapies	Class of CART	Disease	CRS: All grades	CRS: Grade > = 3	Neurotoxicity All	Neurotoxicity Grade = > 3	References
ABECMA	BCMA-BBz	Multiple myeloma	84%	5%	18%	3%	Munshi (2021) [26]
BREYANZI	CD19-BBz	Large B-cell lymphoma	49%	1%	12%	4%	Kamdar (2022) [27]
CARVYKTI	BCMA-BBz	Multiple myeloma	95%	4%	21%	9%	Berdeja (2021) [28]
KYMRIAH	CD19-BBz	Large B-cell lymphoma	58%	22%	26%	15%	Schuster (2019) [29]
TECARTUS	CD19-BBZ	B-cell acute lymphoblastic leukemia	89%	24%	60%	50%	Shah (2021) [30]
YESCARTA	CD19-28z	Large B-cell lymphoma	92%	6%	60%	21%	Locke (2022) [31]

Similarly, an initial survey found an incidence of only 3.48% for HLH/MAS after CAR-T cell therapy between 2016 and 2018 [21]. However, more recent phase I clinical trials of anti-CD22 CAR-T cells reported 32.7% and 35.6% of patients developing HLH, respectively [17, 18]. These findings, coupled with the high morbidity and mortality associated with this syndrome, are drawing increasing attention towards CAR-T cell-related HLH/MAS [21].

CRS, ICANS, and HLH/MAS are understood to be the consequences of CAR-T cell activation in response to tumor recognition, leading to the excessive release of cytokines and danger signals, though this phenomenon is not unique to CAR-T cell therapies and also occurs in other immunotherapies [32–35].

### CAR-T cell-related cytokine release syndrome (CRS)

#### Clinical presentation of cytokine release syndrome (CRS)

CRS is a clinical syndrome that affects multiple organ systems and usually starts from generalized symptoms or signs, such as fever, fatigue, tachycardia, and myalgias. The fever can exceed 105°F/40.5 °C [37]. More severe CRS can present as hypotension, hypoxia, capillary leak syndrome, multiple organ failures, disseminated intravascular coagulation (DIC), and even HLS/MAS [33]. Circulating inflammatory cytokines increase the vascular permeability and third-spacing of fluid, which mimics sepsis but usually with neutropenia [33, 34, 38]. According to the severity of the clinical presentation, CRS can be separated into mild CRS and severe CRS. Constitutional symptoms and/or grade ≤ 2 organ toxicity indicate mild CRS, and severe CRS is characterized by grade ≥ 3 organ toxicity with potentially life-threatening consequences [39–41].

#### Role of pyroptosis and macrophages in CRS

Multiple cytokines are elevated after CAR-T cell infusion, such as interferon-gamma (IFN-γ), tumor necrosis factor alpha (TNF-α), granulocyte–macrophage colony-stimulating factor (GM-CSF), and interleukin 6 (IL-6), but cytokine levels are not always correlated with CRS severity, and their timely monitoring is challenging [5, 39, 41–45]. After antigen binding, CAR-T cells release large amounts of cytokines and perforin/granzymes, which are essential for anti-tumor activity. In addition to caspase 3 activation in the target cells, granzyme A and granzyme B were found to cleave gasdermin D (GSDMD) and E (GSDME), respectively, which are hallmarks of pyroptosis [47–50]. In contrast to apoptosis, which is a non-inflammatory programmed cell death pathway, pyroptosis is a highly inflammatory form of cell death. Cleaved gasdermins release their N-terminal domains, which can insert into the cell membrane and form pores, resulting in the release of pro-inflammatory factors from the dying cells. Thus, high expression of GSDME will lead to preferential pyroptosis, despite both apoptosis and pyroptosis being caspase-mediated [51]. This is consistent with the finding that high GSDME expression is associated with severe CRS [47].

**Fig. 4:**
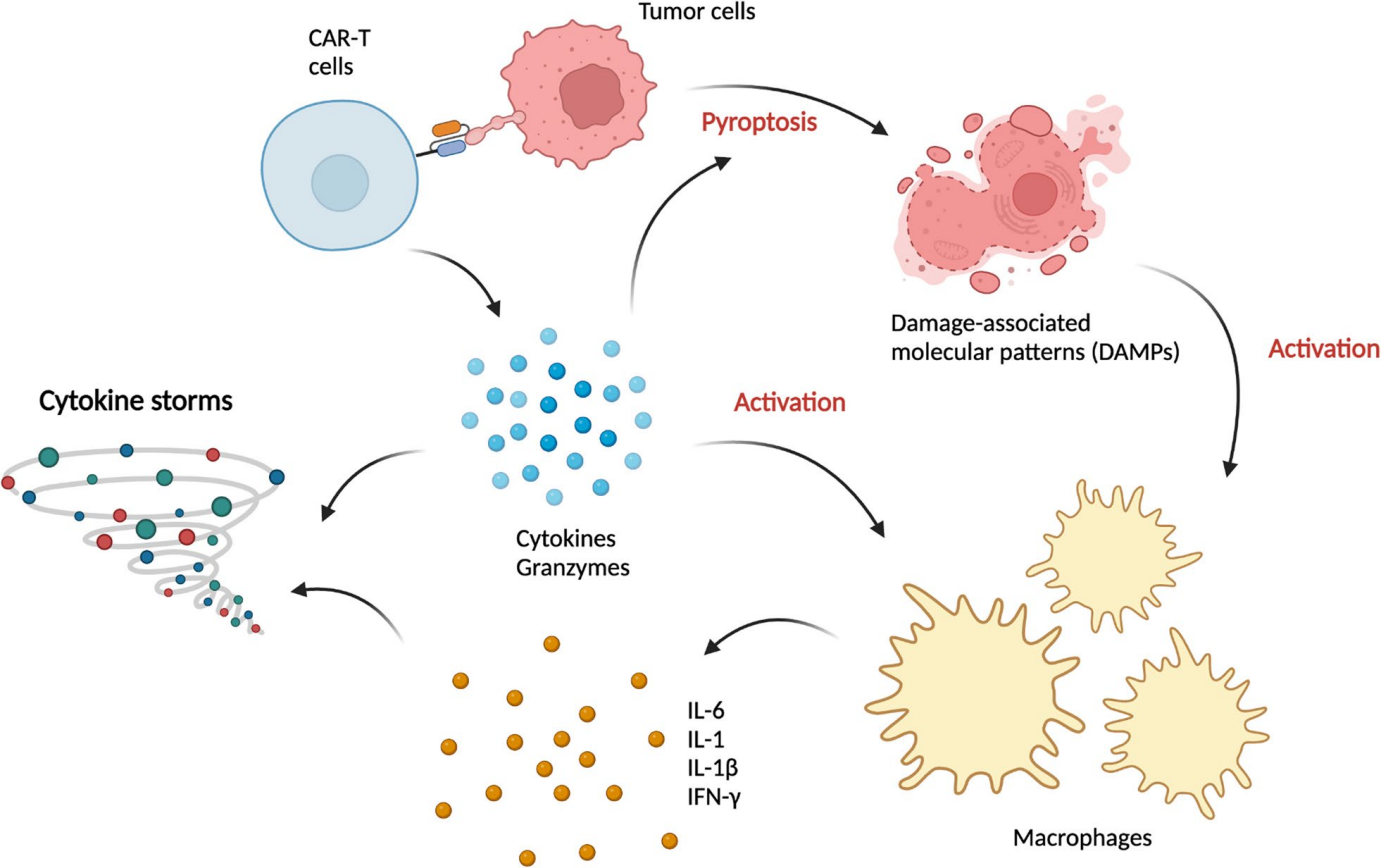
Basic pathophysiology of CRS. Created with BioRender.com

Pyroptotic cells release large amounts of damage-associated molecular patterns (DAMPs), which include heat shock proteins (HSPs) and high­mobility group box 1 (HMGB1) and activate the innate immune system. Here, macrophages and other myeloid cells play a critical role in the pathogenesis of CRS (Fig. 4) [52–54]. HMGB1 binds Toll-like receptor 2 (TLR2) and TLR4 on these cells, activating the interferon regulatory factor (IRF), mitogen-activated protein kinase (MAPK), and NFκB pathways [46]. These pathways trigger the release of systemic cytokines, including interferons, IL-1β, and IL-6 [46, 47, 52, 53]. Activation of TLR2 induces not only the expression and secretion of IL-6 but also the generation of soluble IL-6 receptor (sIL-6R), which enhances the pro-inflammatory properties of IL-6 [55].

In summary, CAR-T cells, which are designed to achieve high anti-tumor efficacy through combinations of T cell-activating signaling domains and high-affinity antigen recognition, inevitably cause the secretion of a large amount of perforin and granzymes [46, 56, 57]. This supraphysiological response may cause excessive pyroptosis, initiating a cascade that leads towards CRS.

#### Role of IL-6 in CRS

IL-6 is well known for its pleiotropic function, including the involvement in B cell and T cell differentiation, bone homeostasis, production of acute-phase proteins, and chronic inflammatory processes in vascular endothelial cells [62, 63]. Early studies in glucocorticoid-resistant graft-versus-host disease (GVHD) indicated its central role in CRS, which motivated the use of tocilizumab, an IL-6 receptor antagonist, for the control of CAR-T cell-related CRS [24, 58]. The effectiveness of tocilizumab was demonstrated later by further studies, and it was approved by the US FDA for the management of CRS in CAR-T cell therapies alongside Kymriah in 2017 [43, 59].

IL-6 can be released by macrophages and other immune-related cells, and it can signal in three different forms: classic signaling through membrane‐bound IL‐6R, trans-signaling in conjunction with the soluble-form of IL-6R, and trans-presentation by dendritic cells [60]. These IL-6/IL-6R complexes bind gp130, which is ubiquitously expressed in various tissues and triggers the activation of Janus kinase (JAK)–signal transducer and activator of transcription (STAT), mitogen‐activated protein kinase (MAPK), phosphoinositide 3‐kinase (PI3K) and YES‐associated protein 1 (YAP1) pathways [60]. Dysregulation of IL-6 can cause tissue damage in autoimmunity and chronic inflammation [61]. In Fig. 4, we summarized the interaction of CAR-T cells, macrophages, and IL-6.

#### Classification of CRS severity and biomarkers

Since fever is the most common hallmark of CRS, the possibility of infection should be considered in all patients with fever after CAR-T cell therapies, and appropriate cultures followed by empiric antibiotics should be initiated [40]. CRS is a systemic syndrome, and one of the most concerning life-threatening complications is cardiac dysfunction, which is likely caused by similar mechanisms as sepsis-related cardiomyopathy [64]. The US National Cancer Institute updated its grading system for CRS to version 5 in 2017. Lee et al. modified the Common Terminology Criteria for Adverse Events (CTCAE) v4.0 to a new grading system in 2014 [40], emphasizing the importance of diagnosis and clinical judgment in determining the appropriate management strategy. The American Society for Transplantation and Cellular Therapy (ASTCT) also proposed new definitions and grading for CRS in 2018, which consider the three major signs: fever, hypotension, and hypoxia [65]. Table 3 provides an overview of these grading systems.

**Table 3:** Summary of grading systems of CRS

NIH CTCAE (Version 5.0)					
Grade	Grade 1	Grade 2	Grade 3	Grade 4	Grade 5
CTCAE (Symptoms and Signs)	Fever with or without constitutional symptoms	Hypotension responding to fluids; hypoxia responding to < 40% O2	Hypotension managed with one pressor; hypoxia requiring ≥ 40% O2	Life-threatening consequences; urgent intervention indicated	Death

Lee’s modified grading system					
Lee’s criteria (Symptoms and Signs)	(i) Symptoms are not life-threatening and require symptomatic treatment only,	(i) Symptoms require and respond to moderate intervention (ii) Oxygen requirement < 40% (iii) Hypotension responsive to fluids or low dose of one vasopressor (iv) Grade 2 organ toxicity	(i) Symptoms require and respond to aggressive intervention (ii) Oxygen requirement ≥ 40% (iii) Hypotension requiring high dose or multiple vasopressors (iv) Grade 3 organ toxicity or grade 4 transaminitis	(i) Life-threatening symptoms (ii) Requirement for ventilator support (iii) Grade 4 organ toxicity (excluding transaminitis)	Death
Treatment recommendation	Supportive care	Supportive care or supportive care + Tocilizumab ± corticosteroids	Supportive care + Tocilizumab ± corticosteroids	Supportive care + Tocilizumab ± corticosteroids	N/A

ASTCT grading system					
Fever	Temperature ≥ 38 °C	Temperature ≥ 38 °C	Temperature ≥ 38 °C	Temperature ≥ 38 °C	Death
With					
Hypotension	None	Not requiring vasopressors	Requiring a vasopressor with or without vasopressin	Requiring multiple vasopressors (excluding vasopressin)	Death
And/Or					
Hypoxia	None	Requiring low-flow nasal cannula or blow-by	Requiring high-flow nasal cannula, facemask, nonrebreather mask, or Venturi mask	Requiring positive pressure (e.g., CPAP, BiPAP, intubation, and mechanical ventilation)	Death

After a retrospective analysis by Davila et al., a clear difference in CRP levels was found between the patients with and without severe CRS, suggesting that patients whose CRP levels exceed 20 mg/dl are at high risk for severe CRS with a sensitivity of 86% and specificity of 100%, and a decrease in CRP was also consistent with the clinical resolution of CRS [43]. However, the correlation between prognosis and cytokine levels is still controversial. Due to underlying chronic inflammation in cancer, constitutively elevated CRP and other acute inflammatory markers make any prediction challenging [40]. Elevation in ferritin was also noted in some patients with CRS after CAR-T cell therapies, but evidence for the utility of these biomarkers is currently insufficient [40].

#### Management of CRS

The grading system is important for determining the appropriate management strategy. For example, compared to supportive management recommended for grade 1 CRS, the management of grade 2 CRS should be decided based on the comorbidities and age, such that individuals at risk of developing more severe complications receive more aggressive treatment, including IL-6 inhibitors and/or corticosteroids [40]. For severe CRS (grade ≥ 3), anti-inflammatory therapies should be applied, which include corticosteroids and anti-cytokine therapies [40, 66]. Early intervention with tocilizumab and/or corticosteroids were shown not to have a negative impact on the anti-tumor potency of CAR-T cells [67]. Considering the mechanism of CRS, anti-IL-6 and anti-IL-1β are likely to provide efficient treatment options for cytokine storms. Tocilizumab, an IL-6 receptor antagonist which prevents IL-6 from binding to both cell-associated and soluble IL-6R, is currently the only US-FDA approved therapy for treating CAR-T cell-associated CRS [68]. CRS is resolved in most patients within 7 days after administration of tocilizumab, and there were no reports of adverse reactions to this drug [68, 69]. A response to tocilizumab is usually observed within hours, but if no improvement of the patient’s condition is seen within 24 h, a second dose or addition of corticosteroids can be considered [40]. Occasionally, some patients subsequently develop signs and symptoms of neurotoxicity after the administration of tocilizumab, which is likely caused by the transient rise of serum IL-6 levels after IL-6 receptor blockade, increasing IL-6 leakage through the blood–brain barrier [40, 70].

Due to the effectiveness of tocilizumab, corticosteroids are considered only a second-line treatment for CAR-T cell-induced CRS. While methylprednisolone or dexamethasone can be administered, dexamethasone showed more efficient penetration through the blood–brain barrier [71]. Corticosteroids are an effective nonspecific anti-inflammatory treatment choice, but some reports also showed ablated effects of CAR-T cells upon steroid use [41, 43, 46, 67]. A retrospective analysis by Strati et al. showed that higher cumulative doses of corticosteroids were associated with significantly shorter progression-free survival (PFS) and overall survival (OS) [72]. Moreover, both prolonged and early use of corticosteroids were associated with shorter OS in this study, suggesting that corticosteroids should be used at the lowest dose and for the shortest duration [72]. However, other studies demonstrated that short-term use of steroids, even at high dose, did not affect treatment outcomes of CAR-T cell therapies [73]. The ZUMA-3 trial also detected a reduced incidence of severe ICANS with early use of corticosteroids [30]. The ZUMA-1 study also confirmed that prophylactic and earlier use of corticosteroids resulted in prevention of severe CRS, as well as a lower rate of severe neurologic events (NEs) [74]. To summarize, corticosteroids are still an effective second-line treatment for CAR-T cell-related CRS, but if future evidence can provide conclusive support, corticosteroids may see prophylactic or early short-term use for severe CRS prevention.

Interestingly, during a national shortage of tocilizumab in 2021, siltuximab, a monoclonal antibody against IL-6, was used as an alternative. In a study of 135 patients, siltuximab used as first-line treatment was comparable to tocilizumab in the rates of CRS, neurotoxicity, ICU transfer and OS, suggesting that siltuximab can be a suitable substitute for tocilizumab [88].

In addition to IL-6, IL-1 is another important cytokine involved in the pathogenesis of CRS. Anakinra, an IL-1 receptor antagonist, has been shown to abolish both CRS and neurotoxicity in mouse models [75]. In severe CRS, both IL-1 signaling and iNOS induction play critical roles, and the ability of anakinra to cross the blood–brain barrier can potentially provide protection from both CRS and neurotoxicity [76, 77]. A phase I clinical trial showed resolution of HLH/MAS-like toxicities after treatment with anakinra without any negative impact on CAR-T cell expansion [17]. Since the use of anakinra is not currently approved by the FDA for CRS, it is not usually administered as a first-line therapy, which might be limiting its efficacy at the moment [46].

TNF-α is another cytokine involved in CRS, especially after anti-BCMA CAR-T cell infusion. In a study by Zhang et al., administration of TNF-α inhibitor (etanercept) was able to resolve CAR-T cell-induced CRS in three patients without a negative impact on the therapeutic response [86]. Moreover, adalimumab (anti-TNF-α) and an anti-IL1β antibody can synergize to prevent endothelial activation by CAR-T cells in vitro [87].

Other targeted immunosuppressive agents can also be considered, including anti-GM-CSF therapies, as well as JAK/STAT and ITK inhibitors. Treatment targeting GM-CSF, such as lenzilumab, might be a promising option, since animal models demonstrated that GM-CSF neutralization caused a reduction in myeloid and T-cell infiltration, and GM-CSF-deficient CART19 cells demonstrated normal functions and improved overall survival in vivo [78]. The ZUMA-19 phase I clinical trial showed that the administration of 1,800 mg lenzilumab before CAR-T cell infusion prevented severe CRS or neurotoxicity, but this trial only had six participants [79]. The involvement of JAK in cytokine signaling motivated the application of JAK inhibitors as CRS treatment [60]. Ruxolitinib is an FDA-approved JAK1/2 inhibitor for myeloproliferative neoplasms (MPN) and steroid-refractory acute graft-versus-host disease (GVHD) [80, 81]. A pilot study showed rapid resolution of CRS symptoms and a decrease of serum cytokines in four patients with CRS treated with ruxolitinib [82]. Reduced levels of multiple cytokines, including IL-6, IL-12, and IFN-γ, were found after itacitinib, a selective JAK1 inhibitor [83]. Dasatinib, a tyrosine kinase inhibitor, was found to inhibit the phosphorylation of CD3ζ and ZAP70, consequently interrupting CAR signal transduction and stopping cytolytic activity, cytokine production, and proliferation of CAR-T cells [84]. This characteristic of dasatinib makes it suitable for emergency use in CRS, but eliminates the anti-tumor effects of CAR-T cells [46, 84]. Ibrutinib, another tyrosine kinase inhibitor, was tested for management of CAR-T cell-related adverse effects in a pilot study with nineteen patients [85]. CD19 CAR T cells with concurrent ibrutinib led to high rates of minimal residual disease-negative response and were well-tolerated with low CRS severity. Among these choices, further studies are still warranted, and there are ongoing clinical trials regarding the effectiveness of these agents.

In conclusion, the current first-line management for CRS after CAR-T cell infusion is tocilizumab, and corticosteroids may be added for severe or tocilizumab-refractory CRS. Further studies in the future might establish the use of corticosteroids as prophylaxis for CRS. Siltuximab may be used as a substitute for tocilizumab. Other agents targeting cytokines and their signaling pathways in CRS could be considered in refractory cases. However, more studies are required to confirm their effectiveness and support their application in CRS after CAR-T cell therapies.

### CAR-T-related neurologic events

Neurotoxicity is the second most common adverse effect of CAR-T cell therapies and is also known as immune effector cell-associated neurotoxicity syndrome (ICANS) [65]. The median onset of ICANS is later than CRS (Fig. 3), but both are etiologically related and there is a significant correlation between severe CRS and grade ≥ 3 neurotoxicity after the infusion of CD19 CAR-T cells. Severe neurotoxicity is also accompanied by higher peak concentrations of CRP, ferritin, and multiple cytokines, as well as systemic vascular dysfunction [89, 91]. CAR-T-related neurologic events are defined as new neurologic signs or symptoms that occur within 1–3 weeks after CAR-T infusion after exclusion of other possible etiologies [89]. Imaging studies, such as CT scans and MRI, are normal in most cases, but EEGs can show diffuse slowing. Laboratory analysis of the cerebrospinal fluid (CSF) can detect blood–brain barrier (BBB) permeability [89]. Certain baseline characteristics are also associated with increasing risk of neurotoxicity, such as higher tumor burden, infused CAR-T cell doses, and pre-existing neurologic disease [89, 91]. Interestingly, ICANS is observed more frequently after T cell therapies targeting CD19 than other antigens, which may be related to the accessibility of the tumor cells, the high expression of CD19, and the resulting extent of T cell activation [89].

#### Pathophysiology of ICANS

Endothelial activation and the breakdown of the blood–brain barrier (BBB) are the major processes leading to ICANS. Angiopoietins 1 and 2 (Ang-1 and Ang-2) are ligands of the Tie-2 receptor with opposing functions in regulating endothelial activation. In non-inflammatory situations, Ang-2 is less abundant than Ang-1, which tends to stabilize endothelial cells and inhibit pro-inflammatory pathways, while Ang-2 is released during inflammation, which causes endothelial activation and microvascular leakage [90]. The Ang-2:Ang-1 ratio is higher among patients developing severe neurotoxicity after CAR-T cell infusion, as well as the concentration of von Willebrand Factor (VWF), which indicates endothelial activation [89]. However, another study found that the imbalance of the Ang-2:Ang-1 ratio was mainly due to decreased levels of Ang-1 [91]. Since platelets are a major producer of Ang-1, this suggests that thrombocytopenia may be one of the primary factors resulting in the activation of endothelia.

During acute neurotoxicity, high protein concentration and leukocyte count can be found in the cerebrospinal fluid (CSF), indicating disruption of the blood–brain barrier (BBB). Among these leukocytes in the CSF, CAR-T cells can be detected by flow cytometry, which are rare in the CSF of patients without neurologic symptoms [89]. However, another study found no correlation between CSF leukocyte or CAR-T count and neurotoxicity grade, casting doubt on their direct involvement in the pathogenesis of ICANS [91]. Instead, cytokine levels in the CSF correlated with psychiatric symptoms, and during the acute phase, levels of IFN-γ, TNF-α, IL-6, and TNFR p55 were comparable between serum and CSF, which suggested that the BBB was not able to prevent the entrance of cytokines into the central nervous system [89, 91, 92].

Other cells involved in ICANS include brain pericytes, which produce IL-6 and VEGF in response to TNF-α and IFN-γ and further amplify endothelial activation and BBB permeability [89, 93]. Macrophage chemotactic protein 1 (MCP1) was also found elevated in CSF during neurotoxicity, which is a chemokine produced by macrophages and microglia, indicating that activation of these cells may also contribute neurotoxicity [91, 94]. Macrophages stimulated by IFN-α2 and IFN-γ could increase the production of quinolinic acid, an endogenous neurotoxin related to seizures and some neurologic disorders [95–97].

In patients with severe neurotoxicity, thrombocytopenia was observed alongside highly elevated von Willebrand Factor (vWF) [89], which is usually stored as ultra-large vWF multimers in Weibel-Palade bodies of endothelial cells and released upon activation [98]. Extracellular vWF unfurls into strings to which platelets attach, causing microthrombi. In addition, IFN-γ, TNF, IL-4, and IL-6 can inhibit the production of ADAMTS13 (a disintegrin-like and metalloprotease domain with thrombospondin type-1 motif, number 13), which is responsible for vWF cleavage [46, 98–100]. Thus, endothelial activation is likely the underlying cause of the consumptive coagulopathy and thrombocytopenia associated with severe ICANS [46, 89].

#### Symptoms and grading systems of ICANS

Signs of CAR-T cell-related neurotoxicity are more diverse than CRS and can range from mild symptoms such as dysgraphia, impaired attention, apraxia, headache, sleep disorder, anxiety, myoclonus, motor dysfunction, and dyscalculia, to more severe symptoms, including encephalopathy, aphasia, delirium, tremors, seizures, and cerebral edema [89, 91].

The ASTCT defines ICANS as a disorder characterized by a pathologic process involving the central nervous system after the infusion of therapy, and thus, their grading system focuses on the evaluation of aphasia, altered level of consciousness, impairment of cognitive skills, motor weakness, seizures, and cerebral edema, excluding other symptoms as less specific [65]. Here, the Immune Effector Cell-Associated Encephalopathy (ICE) score was devised as an objective and reliable screening tool for cognitive function in adult patients. Table 4 summarizes the components of the ICE score and other parts of the ASTCT ICANS grading system [65].

**Table 4:** ICE score, ASTCT grading systems of ICANS and management of ICANS

Immune effector cell-associated encephalopathy (ICE) score	
Orientation	Orientation to year, month, city, hospital: 4 points
Naming	Ability to name 3 objects (e.g., point to clock, pen, button): 3 points
Following commands	Ability to follow simple commands (e.g., “Show me 2 fingers” or “Close your eyes and stick out your tongue”): 1 point
Writing	Ability to write a standard sentence (e.g., “Our national bird is the bald eagle”): 1 point
Attention	Ability to count backward from 100 by 10: 1 point
Scoring: 10, no impairment; 7–9, grade 1 ICANS; 3–6, grade 2 ICANS; 0–2, grade 3 ICANS; 0 due to the unarousable patient and unable to perform ICE assessment, grade 4 ICANS	

ASTCT grading systems of ICANS					
Items	Grade 1	Grade 2	Grade 3	Grade 4	Grade 5
ICE score	7–9	3–6	0–2	0 (unarousable patient and unable to perform ICE assessment)	Death
Depressed level of consciousness	Awakens spontaneously	Awakes to voice	Awakens only to tactile stimulus	The patient is unarousable or requires vigorous or repetitive tactile stimuli to arouse. Stupor or coma	Death
Seizure	N/A	N/A	Any clinical seizure focal or generalized that resolves rapidly or nonconvulsive seizures on EEG that resolve with intervention	Life-threatening prolonged seizure (> 5 min); or Repetitive clinical or electrical seizures without return to baseline in between	Death
Motor findings	N/A	N/A	N/A	Deep focal motor weakness such as hemiparesis or paraparesis	Death
Elevated ICP/cerebral edema	N/A	N/A	Focal/local edema on neuroimaging	Diffuse cerebral edema on neuroimaging; decerebrate or decorticate posturing; or cranial nerve VI palsy; or papilledema; or Cushing's triad	Death

Management					
Grade	Grade 1	Grade 2	Grade 3	Grade 4	Grade 5
Recommended management	Supportive management	Consider corticosteroid use (≤ 10 days)	Corticosteroid use (≤ 10 days)	Corticosteroid use (≤ 10 days)	N/A

While symptoms of ICANS are generally reversible with proper treatment, they can be highly disturbing. Therefore, education and counseling of patients, their families, and medical staff is advised [34].

#### Management of ICANS

A higher risk of ICANS is associated with greater, earlier CAR-T expansion in vivo [28, 101], and higher CAR-T doses as well as severe CRS are risk factors of severe ICANS [28, 89]. According to the criteria of the ASTCT grading system, ICANS is a clinical diagnosis, and daily EEG, MRI, and lumbar puncture can help with differential diagnosis for specific ICANS treatment [65, 102]. EEG should be continued until seizures are resolved [102]. For grade 1 ICANS, supportive treatment is provided, and for ICANS with grades ≥ 2, corticosteroid therapy with rapid taper is indicated [65] (Table 4). Corticosteroids are considered first-line treatment for ICANS, and individuals with seizures can be treated with levetiracetam with or without benzodiazepines for status epilepticus [65, 102]. Dexamethasone is considered the first choice to treat ICANS due to its efficient penetration of the BBB [71]. The effectiveness of corticosteroids (with rapid tapering once ICANS is resolved) was demonstrated in both ZUMA-1 and ZUMA-3 trials [30, 73, 74]. Short-term use of steroids is not likely to have an impact on CAR-T cell efficacy [73]. A duration of corticosteroid treatment of less than 10 days did not alter the patients’ overall outcome or progression-free survival, and no difference was found in the disease response rate [103]. Since tocilizumab is unable to cross the blood–brain barrier, and due to the transient rise of IL-6 serum levels and neurotoxicity often observed following tocilizumab administration, it is not considered a suitable treatment for ICANS [28, 40, 69, 70]. As a result, diagnosis of different types of adverse effects after CAR-T therapy infusion is important to determine management strategies. For example, a finding of grade 1 CRS with concurrent high-grade ICANS would indicate corticosteroids as the appropriate treatment [65]. Further studies may identify new and more specific agents for the treatment of ICANS in the future.

### CAR-T cell-associated hemophagocytic lymphohistiocytosis (carHLH)

Secondary hemophagocytic lymphohistiocytosis (sHLH) is also known as macrophage activation syndrome (MAS), which can not only occur after CAR-T therapies but also after allogeneic and autologous hematopoietic stem cell transplantation (HSCT) [21, 104]. Incidence of HLH/MAS post-HSCT was about 3–4%, but mortality was up to 80% in some studies [21, 104, 105]. Similar to post-HSCT HLH/MAS, the incidence of HLH/MAS post-CAR-T was 3.48% in a survey conducted by Sandler et al. [21]. However, CAR-T cell-associated HLH (carHLH) was found in 35.6% of patients receiving anti-CD22 CAR-T cells in a phase I clinical trial [18].

Diagnosis of sHLH/MAS requires both clincial suspicion and signs of hyperinflammation, which usually includes fever, several-lineage cytopenia, and multi-organ failure. Serum ferritin is used as a biomarker related to disease activity [21, 106–108]. Generally, ferritin levels exceed 1000 ng/ml at the early phase of sHLH/MAS and will uptrend to more than 10,000 ng/ml with concurrent disseminated intravascular coagulation (DIC) [106]. Patients with persistently high ferritin levels had poorer outcome compared to the ones with down-trending ferritin level, with high specificity for disease detection using this metric [106, 107]. Less than 50% down-trending ferritin level, as compared to a 96% or greater decrease, was associated with higher mortality rates, so regular ferritin measurement may be useful for prediction of prognosis [108]. Similar to ferritin, multiple cytokines associated with HLH, such as IFN-γ, IL-8, and MIP-1α, remain persistently elevated in patients with carHLH compared to those with severe CRS [18].

Most studies related to HLH/MAS were based on rheumatological pratice, and HScore or HLH-2004 criteria are used to determine the risk of developing HLH/MAS and criteria of diagnosis, which include both clinical and laboratory features [109]. Neurological dysfunction, acute kidney injury and acute respiratory distress can indicate poor prognosis of HLH/MAS [110]. Due to similar clinical presentations, CRS and HLH/MAS are suspected to belong to a common spectrum, with HLH/MAS being a more severe hyperinflammatory syndrome, which can make HLH/MAS difficult to distinguish from severe CRS [34]. In the study of Lichtenstein et al., all cases with carHLH developed CRS first, and they also found that about 40.4% patients with CRS developed carHLH [18]. The onset of carHLH was 14 days post-infusion, which is later than the median onset of CRS, and severe CRS was associated with a higher risk of developing carHLH.

Challenges in diagnosis and inconsistent reports of carHLH between different studies may be attributed to a lack of uniform diagnosis criteria. Laboratory features commonly used for differential diagnosis are ferritin, fibrinogen, triglycerides, and bone marrow biopsy [21]. Hepatic transaminitis, hyperbilirubinemia, hypofibrinogenemia, hypertriglyceridemia, LDH elevation, and neutropenia are also more commonly found in patients with carHLH compared those without carHLH, although these features might be less specific [18]. Standard carHLH diagnosis criteria were proposed by Neelapu et al., which included peak ferritin levels of > 10,000 ng/ml during the CRS phase, developing into any two of the following: grade ≥ 3 organ toxicities involving the liver, kidney, or lung, or hemophagocytosis in the bone marrow or other organs (Table 5) [34].

**Table 5:** Diagnosis criteria of CAR-T-cell-related HLH/MAS

Ferritin level	A peak serum ferritin level of > 10,000 ng/ml (during CRS phase)
AND	
Clinical and Pathology (any two of the criteria)	Grade ≥ 3 increase in serum bilirubin, aspartate aminotransferase, or alanine aminotransferase levels
	Grade ≥ 3 oliguria or increase in serum creatinine levels
	Grade ≥ 3 pulmonary edema
	Presence of haemophagocytosis in bone marrow or organs based on histopathological assessment of cell morphology and/or CD68 immunohistochemistry

#### Pathophysiology of carHLH

HLH/MAS is a syndrome characterized by cytokine storm, hemophagocytosis and multi-organ damage, which may be caused by defects in normal cytolytic function of NK cells and CTLs [110, 111]. Unlike in CRS, IL-1 appears to be central to HLH/MAS, and excellent clinical responses were observed to IL-1 blockade [110, 112, 113]. IL-1β levels were particularly high among those patients with carHLH, which motivated the use of anakinra, an IL-1 receptor antagonist, for management of this syndrome [18]. Furthermore, the majority of carHLH patients received tocilizumab with significant improvement of CRS but still continued to develop carHLH, supporting a dominant role of IL-1 rather than IL-6 [18]. However, the exact mechanism is still poorly understood.

Genetic disposition and inability to clear infectious antigen or malignancy may all be the original cause of this syndrome. In the bone marrow of carHLH patients, higher T cell to NK cell ratios were found, alongside higher peak expansion and diminished contraction of CAR-T cells in peripheral blood [18]. Higher baseline levels CD3^+^ and CD8^+^ cells and relatively lower frequencies of NK cells were also found in the peripheral blood of patients who later developed carHLH [18]. Relative pre-infusion NK cell lymphopenia became more profound in carHLH, which likely contributed to the inability of controlling CAR-T cell proliferation and expansion [18, 118], since NK cell activity is known to limit CD8^+^ T cell immunity [119]. In a mouse model, NK cell-mediated cytotoxicity was found to be able to reduce HLH-like manifestations and limit activity of cytotoxic T lymphocytes [120]. Dendritic cells sometimes expressed CD22 in rare cases of leukemia, which may contribute to the higher incidence of carHLH in trials of CD22 CAR-T therapies, but the correlation and further mechanism need more studies in the future [18, 121]. Excessive T cell expansion is also known to be the cause of other secondary hyperinflammatory responses [122].

#### Management of sHLH/MAS and carHLH

Aggressive immunosuppression is required for management of HLH/MAS and in combination with treatment targeting the etiological factors [21]. Due to clinical overlap of criteria between CRS and CAR-T cell-related HLH/MAS, it is recommended to administer standard treatment for CRS, including anti-IL-6 therapy and corticosteroids [34]. According to the study by Neelapu et al*.*, if no improvement is seen within 48 h, etoposide for refractory HLH should be considered, and intrathecal cytarabine may be used for HLH-associated neurotoxicity [34]. Carter et al. proposed using methylprednisolone and intravenous immunoglobulin (IVIG) as first line treatment, followed by anakinra as second-line treatment for sHLH/MAS, with application of etoposide regarded as a third choice [110]. However, management strategies may differ depending on the etiology of HLH/MAS. For example, IVIG and rituximab are used for sHLH/MAS triggered by EBV or EBV-driven malignancies [21, 116, 117]. On the other hand, anakinra, an IL-1 receptor antagonist, is now used in refractory sHLH/MAS and systemic juvenile idiopathic arthritis (sJIA)-triggered sHLH/MAS [21, 114, 115]. Anakinra has also been successfully used for treatment of carHLH [18, 123, 124]. Other agents targeting cytokines involved in carHLH might be taken into consideration for future application. For example, emapalumab, an FDA-approved monoclonal antibody targeting IFN-γ for refractory HLH, might be suitable for use in carHLH, but the possible impact of emapalumab on CAR-T cell efficacy should be taken into consideration [18, 122].

According to the ASTCT, the considerable overlap between CRS and carHLH makes the proper diagnosis difficult and not always separable. Combined with early reported low incidence, carHLH was thus regarded as a severe complication of CRS rather than its own syndrome, and a separate grading system may not be required [65, 125, 126]. However, considering the high occurrence of carHLH in some recent clinical trials, its high morbidity and mortality, as well as the successful application of anakinra rather than tocilizumab in carHLH, further studies will be helpful to establish new standards for carHLH diagnosis and management protocols [18].

### Other CAR-T cell-related adverse effects

Antigens targeted by CAR-T cell therapies can be expressed in normal tissues, causing on-target off-tumor toxicity. The most common target of current CAR-T cell therapies is CD19 which is expressed in B cells. Therefore, eradication of B-lineage cells is a common side effect of anti-CD19 CAR-T, resulting in B cell aplasia and hypogammaglobulinemia [9]. Decreasing blood cell counts, such as thrombocytopenia, anemia, neutropenia and other leukopenia are also common, with an incidence between 30% and 90% [6, 19, 29, 127, 128]. Patients with persistent cytopenia are susceptible to infectious complications, especially opportunistic infections such as herpes zoster and Pneumocystis jirovecii pneumonia [6, 34, 129]. The ASCO guideline recommends use of granulocyte-colony stimulating factor (G-CSF) to treat CAR-T cell-related neutropenia instead of GM-CSF, since GM-CSF may aggravate CRS [130, 131]. Intravenous immunoglobulin is recommended to manage B cell aplasia and hypogammaglobulinemia [132].

Graft-versus-host disease (GVHD) after autologous CAR-T therapies infusion is rare. According to retrospective studies, the only GVHD observed was slowly worsening pre-existing chronic GVHD, and no new-onset acute GVHD after infusion of anti-CD19 CAR-T cells was found [20, 25, 133, 134]. The dose of CAR-T cell infusions at 10^6^ to 10^7^/kg is also below the suggested threshold for standard donor lymphocyte infusions (DLIs) of 10^7^ T cells/kg, where potent graft-versus-leukemia (GVL) effects are observed rather than GVHD [20, 135]. However, as allogeneic CAR-T cell therapies are developed, evaluating the risk of GVHD remains important.

### Other prevention and management of toxicities

Reducing the CAR T-cell dose in patients with a high tumor burden can decrease the risk of severe CRS, which proved effective in B-ALL patients without impairing efficacy [136, 137]. However, careful management and calculation is required due to the narrow therapeutic window [137], and increased relapse rates raised concerns of impairing the long-term prognosis [136]. Instead, Frey et al. proposed split-dosing, and their clinical trial demonstrated that a fractionated dosing scheme may be personalized according to the patient’s risk of CRS and disease burden, since 2-year survival rates improved in the high-dose fractionated infusion group [46, 138, 139].

Another approach is to change the structure of the CAR. A new CD19 single-chain variable fragment (scFv) with lower affinity to CD19 but higher efficacy showed a promising safety profile with no severe CRS, and only grade 1–2 CAR-T cell-related neurotoxicity [140]. Compared to CD28 costimulatory domains, CAR T-cells with 4-1BB costimulatory domains also tend to have lower toxicity due to more mild but persistent tumor killing, compared to the more rapid action of CD28-based CAR-T cells [46, 91, 141]. However, novel CAR designs with balanced efficacy, persistence and toxicity profiles are still under active investigation.

For improved control over CAR-T cell activity, various molecular switches have been proposed, such as reversible small molecule-induced dimerization-based OFF- and ON-switches using Bcl-XL inhibitor A1155463 or lenalidomide [142, 143]. Another option is the development of conditional antigen-binding domains, where antigen recognition can be modulated using FDA-approved small molecule drugs, as was demonstrated with methotrexate [144].

On the other hand, irreversible switches allow for permanent removal of CAR-T cells in case of emergency. Addition of cell surface antigens to the CAR-T cells can allow for depletion through FDA-approved monoclonal antibodies such as rituximab or cetuximab, while expression of inducible caspase 9 can force CAR-T cells to undergo apoptosis upon administration of a small molecule [145–147]. However, both reversible and irreversible switches are still under pre-clinical and clinical investigation. Several factors need to be taken into consideration, such as the safety and bioavailability of small molecules, timing of clinical use, the reaction time of the switches in the human body, and the overall impact on efficacy of CAR-T cells [46].

## Conclusion

In this review, we summarized the major adverse effects of CAR-T cell therapies, including CRS, ICANS, and carHLH, as well as their current management protocols. As CAR-T cells are applied more widely in the clinic, future studies will improve our understanding of their impact on the human body and provide clinicians with more reliable standardized diagnosis and treatment algorithms. Novel findings may also help researchers develop safer CAR-T cell products with improved CAR designs.

## Data Availability

Not applicable.

## Abbreviations

CAR-T cell therapies: Chimeric antigen receptor T cell therapies
CRS: Cytokine release syndrome
ICANS: Immune effector cell-associated neurotoxicity syndrome
carHLH: CAR-T cell associated HLH
LBCL: Large B cell lymphoma
MCL: Mantle cell lymphoma
ALL: Acute lymphoblastic leukemia
BCMA: B cell maturation antigen
CARs: Chimeric antigen receptors
TCR: T cell receptor
HLH/MAS: Hemophagocytic lymphohistiocytosis/macrophage activation syndrome
irAEs: Immune-related adverse events
DIC: Disseminated intravascular coagulation
IFN-γ: Interferon-gamma
TNF-α: Tumor necrosis factor alpha
GM-CSF: Granulocyte–macrophage colony-stimulating factor
IL-6: Interleukin 6
GSDMD: Gasdermin D
GSDME: Gasdermin E
DAMPs: Damage-associated molecular patterns
HSPs: Heat shock proteins
HMGB1: High-­mobility group box 1
TLR: Toll-like receptor
IRF: Interferon regulatory factor
MAPK: Mitogen-activated protein kinase
sIL-6R: Soluble IL-6 receptor
GVHD: Graft-versus-host disease
JAK–STAT: Janus kinase–signal transducer and activator of transcription
MAPK: Mitogen‐activated protein kinase
PI3K: Phosphoinositide 3‐kinase
YAP1: YES‐associated protein 1
CTCAE: Common terminology criteria for adverse events
ASTCT: The American Society for Transplantation and Cellular Therapy
PFS: Progression-free survival
OS: Overall survival
NEs: Neurologic events
MPN: Myeloproliferative neoplasms
CSF: Cerebrospinal fluid
BBB: Blood–brain barrier
Ang-1 and Ang-2: Angiopoietins 1 and 2
VWF: Von Willebrand factor
MCP1: Macrophage chemotactic protein 1
ADAMTS13: A disintegrin-like and metalloprotease domain with thrombospondin type-1 motif, number 13
ICE: Immune effector cell-associated encephalopathy
sHLH: Secondary hemophagocytic lymphohistiocytosis
HSCT: Hematopoietic stem cell transplantation
IVIG: Intravenous immunoglobulin
G-CSF: Granulocyte-colony stimulating factor
GM-CSF: Granulocyte–macrophage colony-stimulating factor
GVL: Graft-versus-leukemia
